# The Mediterranean Diet, a Rich Source of Angiopreventive Compounds in Cancer

**DOI:** 10.3390/nu11092036

**Published:** 2019-08-31

**Authors:** Beatriz Martínez-Poveda, José Antonio Torres-Vargas, María del Carmen Ocaña, Melissa García-Caballero, Miguel Ángel Medina, Ana R. Quesada

**Affiliations:** 1 Department of Molecular Biology and Biochemistry, Faculty of Sciences, University of Málaga, Andalucía Tech, 29071 Málaga, Spain; 2 IBIMA (Biomedical Research Institute of Málaga), 29071 Málaga, Spain; 3 Laboratory of Angiogenesis and Vascular Metabolism, VIB Center for Cancer Biology, VIB, 3000 Leuven, Belgium; 4 Laboratory of Angiogenesis and Vascular Metabolism, Department of Oncology, Leuven Cancer Institute, KU Leuven, 3000 Leuven, Belgium; 5 CIBER of Rare Diseases, Group U741 (CB06/07/0046), 29071 Málaga, Spain

**Keywords:** angiogenesis, cancer, chemoprevention, angioprevention, Mediterranean diet, bioactive compounds

## Abstract

Diet-based chemoprevention of cancer has emerged as an interesting approach to evade the disease or even target its early phases, reducing its incidence or slowing down tumor progression. In its basis in the essential role of angiogenesis for tumor growth and metastasis, angioprevention proposes the use of inhibitors of angiogenesis in cancer prevention. The anti-angiogenic potential exhibited by many natural compounds contained in many Mediterranean diet constituents makes this dietary pattern especially interesting as a source of chemopreventive agents, defined within the angioprevention strategy. In this review, we focus on natural bioactive compounds derived from the main foods included in the Mediterranean diet that display anti-angiogenic activity, as well as their possible use as angiopreventive agents.

## 1. Angiogenesis and Cancer

### 1.1. Angiogenesis as a Regulated Process

Angiogenesis, the neoformation of vessels from an existing vascular bed, is an important process during development; however, in adulthood most of the blood vessels remain largely quiescent, with some physiological exceptions, such as wound healing, ovulation and tissue repair. Angiogenic phenomena are crucial for normal physiological functions and must be carefully controlled to maintain healthy conditions. Therefore, it is not surprising that a deregulated angiogenesis plays an essential role in multiple pathological situations, including atherosclerosis, diabetic retinopathy, rheumatoid arthritis, macular degeneration, psoriasis, tumor growth, metastasis, and chronic inflammation [1].

The multistep process of angiogenesis starts with the vasodilation and increased permeability of existing vessels, endothelial cell activation and proliferation in response to angiogenic factors. Thereafter, the degradation of the capillary wall by extracellular proteinases occurs, followed by migration of endothelial cells, formation of new capillaries, and finally, the interconnection of the new vessels (anastomosis) and their stabilization by recruitment of pericytes [2]. All these steps are controlled by a tight balance, both spatially and temporally, between activators (growth factors, i.e., vascular endothelial growth factor (VEGF), basic fibroblast growth factor (bFGF), platelet-derived growth factor (PDGF), a plethora of cytokines, bioactive lipids, matrix-degrading enzymes, and a number of small molecules) and inhibitors (angiostatin, interferons, endostatin, IL-12 and retinoids) that execute their function through different signaling pathways [3].

### 1.2. Angiogenesis as a Hallmark of Cancer

Cancer represents a leading cause of death in the developed world. Although massive efforts and investments have been made in cancer therapy to successfully treat localized tumors, medicine is nonetheless often helpless in the treatment of metastatic processes. Despite the huge diversity of oncologic diseases, all of them share some fundamental features, pointed out by Hanahan and Weinberg as “hallmarks of cancer” [4,5]. Interestingly, one of them is angiogenesis induction, since a persistently activated and deregulated angiogenesis is essential for tumor growth and metastasis. It is widely accepted that proliferating tumor cells need host support, including the connection of the so-called “angiogenic switch”. Activation of angiogenesis can occur at any step of the tumor progression and depends on the type of tumor and its microenvironment. For instance, many tumors start growing in an avascular phase until reaching a steady state within the proliferating cells. At this stage, the “angiogenic switch” activates endothelial cells to undergo a series of phenotypic changes to finally differentiate into a new vessel. Angiogenesis is therefore a rate-limiting step in progression to solid tumor malignancy. Blood vessels supply nutrients and oxygen, and serve as a route for the elimination of waste, contributing to exponentially enhance the tumor growth. Additionally, the new vasculature also provides a pathway for tumor cells to escape from the primary tumor, invade nearby tissues, move throughout the body, and colonize distant organs, giving rise to metastasis [6].

Tumor angiogenesis significantly differs from physiological angiogenesis. The newly-formed vasculature is aberrant, with altered interactions between endothelial cells and pericytes, abnormal blood flow, and increased permeability—all due to a chaotic and poorly-regulated expression of pro- and antiangiogenic factors. As a consequence, tumor vessels are often disorganized, incomplete, lacking structural integrity, and prone to collapse, resulting in areas of inadequate perfusion and transient hypoxia [7].

Angiogenesis has been defined as an “organizing principle” in biology, allowing connections between unrelated phenomena. Favoring therapies initially designed for the treatment of cancer could be used to treat other non-neoplastic angiogenesis-dependent diseases, including age-related macular degeneration, some retinopathies, psoriasis, or rheumatoid arthritis, among others [8].

### 1.3. Antiangiogenic Therapies in Cancer

The early hypothesis by Judah Folkman in 1971 that tumor dormancy could be maintained by preventing neovascularization of microscopic cancers could not be clinically validated until 2004, when the first antiangiogenic drug received the U.S. Food and Drug Administration (FDA) approval for the treatment of cancer patients [9,10]. Although many questions still remain unanswered, accumulating clinical evidence of antiangiogenic therapies in extending survival in cancer patients make antiangiogenesis one of the most promising anticancer targets [11]. Antiangiogenic inhibitors are unique cancer-fighting agents that can block the growth of blood vessels that support tumor growth rather than interfering with the growth of tumor cells themselves. Over the last decades, angiogenesis has become an appealing target in cancer therapy, being of great interest in the fields of pharmacology and drug discovery. Angiogenesis inhibitors can interfere with one or various steps of the blood vessel growth [10]. This has favored a continuous growth of antiangiogenic drugs in the cancer field, with a myriad of molecules being evaluated in preclinical studies and several hundred reaching clinical trials [12]. Importantly, several compounds have been already approved by the FDA, and they are registered for the clinical treatment of different tumors (Table 1). This list includes humanized antibodies such as bevacizumab or ramucirumab, fusion proteins such as aflibercept, and low molecular weight molecules, including sorafenib, sunitinib, or vandetanib, among others (recently reviewed in [13,14,15]).

Clinical data have shown that angiogenesis inhibitors appear to be most effective when used in combination with other antiangiogenic or traditional anticancer therapies [16], probably due to a “normalization” of tumor vasculature that could help drugs to penetrate the mass and to function more effectively [17]. Nowadays, the use of antiangiogenic strategies is being actively explored to increase the effectiveness and diminish the risk of immune-related adverse effects of immunotherapy, a major therapeutic modality that is revolutionizing the treatment of cancer [18].

## 2. Angioprevention

In the last decades, many research efforts have focused on cancer chemoprevention, a concept firstly defined by Michael B. Sporn in 1976 as the use of natural, synthetic, or biologic agents to prevent, suppress, or reverse tumor progression [19]. Whereas early detection of cancer is a broadly accepted approach, cancer chemoprevention is still a matter of debate in the scientific community, mainly due to the controversial results obtained in some reported preventive interventions [20]. Cancer chemoprevention approaches are based in the interference of one or several of the hallmarks of cancer-using drugs that could slow down the initiation and progression of tumors. In this context, the concept of angioprevention, firstly coined by Adriana Albini and colleagues, arose as the prevention of cancer by inhibition of tumor angiogenesis [21]. Angioprevention applies angiogenesis inhibition to those predisposing conditions, including chronic inflammation, pre-neoplastic or hyperplastic lesions, and microscopic tumors, so that modulation of the tumor microenvironment could help the host defense systems to more efficiently avoid the development of clinically detectable tumors [22].

**Figure 1:**
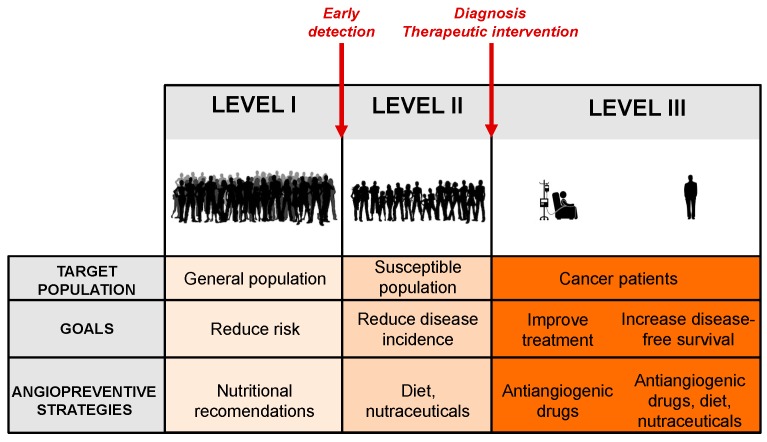
The three levels of cancer chemoprevention. The figure represents the three levels at which cancer preventive action could be implemented, showing the targeted population, the main goals, and the angiopreventive strategies at each level.

There are different levels at which the cancer preventive action could be implemented, depending on the targeted population (Figure 1) [23].

The primary prevention stage is focused on decreasing the incidence of disease in a broad healthy population, mainly by reducing the exposure to carcinogenic factors or by increasing the individuals’ resistance to them. Possible interventions include nutritional recommendations, dietary supplements, and natural inhibitors of angiogenesis, which could help to stop early events in tumorigenesis. The primary preventive agents group also includes all supplements able to induce the intrinsic properties that suppress blood vessel formation by endogenous angiogenesis inhibitors present within the host microenvironment (endostatin, thrombospondin-1, angiostatin, and many others) [2].

Secondary prevention is directed at reducing the mortality from a particular cancer in higher-risk populations, with actions focused on early detection and treatment in the subclinical stages of the disease before symptoms appear. The high-risk population is composed of individuals with genetic abnormalities, usually associated with their lifestyle, specific syndromes, or family history. Improvement in the screening programs and the diagnostic tests will facilitate the early detection of tumors or pretumoral lesions. Inhibitors of angiogenesis may provide a valuable tool to prevent the growth of undetectable primary tumors in this population, a concept that was implicit in Judah Folkman’s visionary idea [9]. For secondary prevention of cancer, a number of natural or synthetic antiangiogenic compounds are available, including fumagillin, fumagillol, genistein, or difluoromethylornithine, among many others [10]. Because of the long-term nature of the earlier preventive strategies, these compounds should be fulfilled with some premises, namely minimal toxicity, low cost price, and capability to promote the physiological anti-tumoral responses of the tumor microenvironment [22].

Tertiary prevention is directed to cancer patients in more advanced stages of the disease and is aimed at improving the oncologic treatment results, their survival rate, and their quality of life. Preventive actions at this level include prevention of recurrence of a pre-existing cancer and the development of new secondary tumors. In this scenario, an aggressive and sustained chemopreventive approach may be required, as tumor cell dissemination is likely to exist. Tertiary prevention could include antiangiogenic cancer agents already approved by the regulatory agencies (Table 1), and others under clinical trials, used either along or after the treatment in order to prevent a relapse. In any case, more sustainable and less aggressive antiangiogenic strategies, such as those used for primary and secondary prevention, could also be of application for cancer patients in order to halt the growth of those undetectable microscopic metastasis that could remain after primary tumor resection.

Increasing evidence supports the angioprevention approach in preclinical models, as well as in epidemiological and clinical intervention studies in humans [24]. The availability of antiangiogenic molecules in dietary sources suggests that a rationally designed antiangiogenic diet could be a natural and cost-effective strategy for cancer prevention [25].

## 3. The Mediterranean Diet: A Cultural Heritage of Humanity

The influence of diet and diet components in health is currently an open study question. Among the known dietary models, the Mediterranean diet (MD) stands out. In as early as 1980, Ancel Keys reported in his Seven Countries Study that the MD was associated with lower risk of major chronic diseases and highest life expectancies [26]. Extended from centuries in the civilizations along the Mediterranean coast, the MD is mainly characterized by the general intake of fruits and vegetables, legumes, cereals, dried fruits, and nuts, together with a low consumption of red meat, low/moderate intake of dairy products, and moderate/high consumption of fish, seasoned by the daily intake of a low quantity of red wine with meals [27]. Although specific MD components exhibit some differences depending on the country, all the observed variants share a common component, that is, the use of virgin olive oil as main addition fat. However, diet components are not the only elements that define the MD, as this ancient dietary pattern—transmitted through generations—includes social and cultural aspects that make it a marker of identity of people who live in the Mediterranean basin, up to the point that the MD was included in 2013 in the Representative List of the Intangible Cultural Heritage of Humanity of UNESCO (United Nations Educational, Scientific and Cultural Organization) [28].

Despite the extended and ancient tradition of the MD in Mediterranean countries, in the last decades, the adherence to this diet has experienced a marked decline, turning to a dietary pattern closer to the western diet model (essentially the nemesis of MD, mainly characterized by the high intake of saturated fats, highly processed foods, regular consumption of red meat, high presence of added-sugar products, and low presence of vegetables, fruits, and fiber [29,30]). Suggested explanations of this so-called “westernization” process point to a rise in the availability of non-Mediterranean food products, together with socioeconomic factors such as the increasing prices of typical products of the MD [31,32]. Unlike this observed negative tendency, the evidence of the health benefits derived from the MD has sparked the interest of the scientific community. Indeed, MD adherence is related to a lower incidence of cardiovascular diseases, as was firstly reported in the Seven Countries Study and confirmed later by many other studies [33,34,35,36,37]. Besides its cardioprotective effects, MD has been associated with a lower risk of certain types of cancer and a decreased total cancer mortality [38,39,40,41], although a higher number of studies are needed to undoubtedly establish this relationship. Indeed, a recent exhaustive umbrella review of meta-analyses of observational studies and randomized trials evidenced that adherence to MD is related to a reduction in cancer incidence, with its role in overall cancer mortality still weakly suggested by current data [42].

The precise molecular mechanism responsible for the effects of MD in health is currently under research. At a molecular level, MD components include a high number of bioactive compounds, with it being difficult to define the precise contribution of any of them. In general, it has been suggested that the combination of components of the diet essentially exert a complex common role in reducing inflammation and oxidative stress, therefore counteracting the progress of several malignancies, including cardiovascular diseases and cancer [43]. It is noteworthy that MD adherence has revealed a protective role against these and many other of the most prevalent diseases [42], connecting this dietary pattern to chemoprevention. The cardioprotective role of MD is rather well defined, since its beneficial influence on traditional atherosclerotic and cardiovascular risk factors has been proven [44,45,46,47]. In contrast, the chemopreventive effect of this dietary pattern on cancer is still misunderstood, although encouraging studies point to the risk-reducing effect of the MD on certain types of cancer, as is the case of breast and colorectal cancer [39,48,49,50,51]. In this context, angioprevention is an important concept to take into account, since angiogenesis prevention through bioactive compounds present in the MD components could explain in part the chemopreventive effect of this diet model in cancer. By extension, angioprevention derived from the MD would be interesting in the prevention of other angiogenesis-dependent diseases, such as diabetic retinopathies or atherosclerosis. In this review, we propose an angiopreventive role of some of the most distinctive components of MD, based on their content of bioactive compounds exhibiting antiangiogenic properties.

## 4. Olive Oil as a Source of Antiangiogenic Molecules

Despite the differences in the components of MD depending on the country, the use of olive oil in cooking and seasonings could be considered the most remarkable common denominator of the diet. It is noteworthy that the health effects of olive oil in several pathologies have been long recognized [52]. Olive oil is defined as oil extracted from the fruit of olive trees (*Olea europaea sativa*). Depending on the degree of processing, olive oils can be classified into different categories. Virgin olive oils are obtained solely by mechanical means that do not lead to alterations in the oil, with extra virgin olive oil being the one of highest quality. Oils simply labelled as “olive oil” have normally been subjected to a refinement process [53].

Olive oil contains a complex mixture of different types of compounds, whose proportion will vary depending on the type of olive, maturation, growing conditions, storage, extraction method, and refinement degree [53,54,55]. In general, olive oil components fall into the saponifiable fraction (98–99% w/w) or the unsaponifiable fraction (1–2% w/w). The saponifiable fraction consists mainly of triglycerides, although diglycerides, monodiglycerides, and free fatty acids are also found in a much smaller proportion. The main triglyceride fatty acid in this fraction is oleic acid, representing 55–83% of the total fatty acid content. In the unsaponifiable fraction, a greater diversity of compounds are found, responsible for stabilizing and protecting the olive oil integrity, preserving its organoleptic characteristics [56]. In this fraction, mainly hydrocarbons, tocopherols, pigments, sterols, triterpene acids, volatile compounds, and phenolic compounds are included. Among them, phenolic compounds of olive oil (including simple phenols, flavonoids, secoiridoids, and lignans) have shown interesting biological activities [55,57]. Extra virgin olive oil shows the highest content of phenolic compound, which are almost absent in refined oils as they are lost during the refinement process [58].

**Figure 2:**
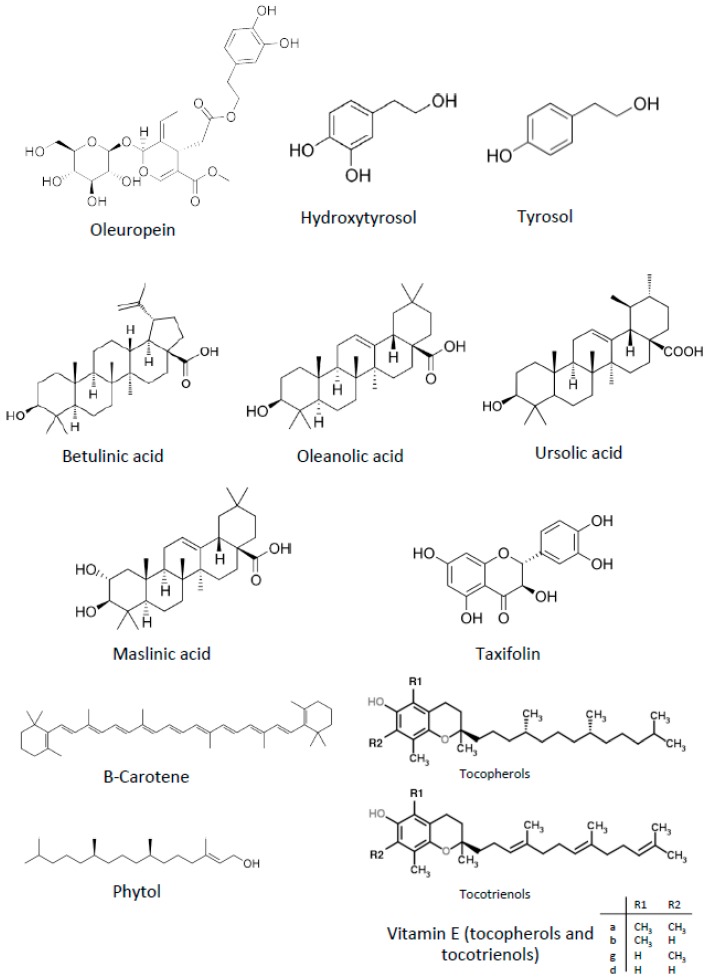
Chemical structures of antiangiogenic compounds present in extra virgin olive oil.

Although traditionally the health effects of olive oil were attributed to its high content in oleic acid, nowadays a high number of scientific studies has demonstrated that these effects must be also attributed to some minority compounds of olive oil, especially those included in the phenolic fraction [59]. Among other multiple biological activities, antiangiogenic properties have been reported for some compounds present in the olive oil, shown in Figure 2 and presented below.

### 4.1. Oleuropein

Oleuropein, a secoiridoid present in olive oil, has been proposed to be a chemopreventive agent, based on its capability to modulate several oncogenic signaling pathways [60]. Regarding angiogenesis, it inhibits VEGFR-2 autophosphorylation at specific sites (Tyr951, Tyr1059, Tyr1175 and Tyr1214), suppressing the VEGF-induced proliferation and migration of macrovascular and microvascular human endothelial cells, as well as their morphogenic differentiation into tubular-like structures in Matrigel [61]. The antiangiogenic activity of this compound could be associated with the inhibition of PMA-induced COX-2 expression, and the decrease in MMP-9 protein release and gelatinolytic activity [62].

During olive ripening, the enzyme β-glucosidase transforms oleuropein into oleuropein aglycone, which by hydrolysis renders elenolic acid and hydroxytyrosol, an interesting bioactive compound that is discussed below.

### 4.2. Hydroxytyrosol

Hydroxytyrosol (2-(3,4-dihydroxyphenyl) ethanol) and tyrosol (2-(4-hydroxyphenyl) ethanol) are the most abundant simple phenols contained in olive oil, also being the major phenolic constituents of wine. Both compounds derive from the natural hydrolysis of the aglycone form of oleuropein and ligstroside, respectively, during olive maturation. In addition to other biological activities, including antitumoral, antiinflammatory, cardio-, and neuroprotective activities, hydroxytyrosol has been shown to inhibit angiogenesis in vitro and in vivo [63,64,65,66,67]. In vitro assays evidenced that hydroxytyrosol is able to inhibit the endothelial cell growth, migration, and tubular-like structures formation. The effect on the endothelial proteolytic balance was demonstrated by a decrease in the release of MMP-2 and MMP-9 by endothelial cells. Ex vivo (rat aortic ring) and in vivo (CAM) angiogenesis assays supported the antiangiogenic activity of hydroxytyrosol [66], which could be related to the inhibition of COX-2 and VEGFR-2 phosphorylation [61,67]. Recently, the antiangiogenic activity of synthetic molecules derived from hydroxytyrosol has been described, showing that chemical modifications in the original molecule may improve the bioactivity of the natural compound, therefore reinforcing their utility as angiopreventive compounds [68]. In contrast with the antiangiogenic activity described for hydroxytyrosol, the closely related compound tyrosol, also present in olive oil, did not show capability to inhibit angiogenesis in vitro [66].

### 4.3. Triterpene Acids

Pentacyclic triterpenes, such as betulinic, oleanolic, ursolic, and maslinic acids, are present in olives, olive tree leaves, and virgin olive oil. There is a large amount of literature that clearly illustrates the significant anti-neoplastic effects of triterpenes, exhibiting multiple biological and pharmacological properties.

Betulinic acid inhibited in vitro the bFGF-induced invasion and the tubular-like structure formation of bovine aortic endothelial cells (BAECs) [69]. This activity has been related to the activation of the proteasome-dependent degradation of the transcription factors, specifically protein Sp1, Sp3, and Sp4, which also regulate VEGF expression [70].

Oleanolic acid, also present in several traditionally-used plants used to treat cancer, is considered to be a chemopreventive compound, probably due to its capability to suppress multiple molecular targets involved in both the development and progression of cancer [71]. Oleanolic acid inhibits angiogenesis in vivo and in vitro via suppression of STAT3 and Hedgehog pathways, which could be one of the underlying mechanisms of its antitumoral effect [72]. Inhibition of VEGFR-2 signaling pathway has been recently linked to the antiangiogenic activity of this compound [73].

Ursolic acid antitumor activity has long been recognized, probably being responsible for the anticancer properties of some plants used in Chinese traditional medicine. The antiangiogenic activity of ursolic acid was evidenced by the early observation that it interfered several steps of the angiogenic process in vitro [74], later being characterized as an inhibitor of multiple signaling pathways controlling cancer growth and angiogenesis [75], including that of HIF-1α [76]. In addition, studies of the ursolic acid biodisponibility after oral administration in mice, and the toxicity, pharmacokinetic, and pharmacodynamics of liposomal ursolic acid in humans have shown promising results, supporting its use in cancer chemoprevention [77]. Extensive effort is being devoted to the synthesis of more effective ursolic acid derivatives with improved chemopreventive potential [78,79].

Maslinic acid, aside from its anti-oxidant, anti-inflammatory, and anti-viral activities, exhibits significant anticancer and antiangiogenic properties that could be mediated by the decrease in the expression levels of NF-κB-regulated genes. These include genes involved in tumor cell proliferation (Cyclin D1, COX-2, and c-Myc), apoptosis (Survivin, Bcl-2, Bcl-xl, XIAP, and IAP-1), invasion (MMP-9 and ICAM-1), and angiogenesis (VEGF) [80].

The pharmacological relevance of all the aforementioned activities of the natural triterpenes found in olive oil has fueled the interest either in the synthesis of new synthetic derivatives with higher potency and efficacy, or in the development of new delivery approaches that could increase the therapeutic potential of those compounds [81,82], which could be better used in the angioprevention of cancer.

### 4.4. Other Antiangiogenic Compounds Present in Olive Oil

The antineoplastic properties of some other compounds found in olive oil have been described, many of them also exhibiting antiangiogenic activities. They include flavonoids, carotenoids, vitamin E, diterpenoids, and sterols (Figure 2).

Taxifolin, a flavanonol (flavonoid) present in olive oil, inhibits the in vitro formation of tubular-like endothelial cell structures on Matrigel, as well as the in vivo formation of new blood vessels in the CAM and the murine dorsal skinfold chamber models [83]. Mechanistically, the antiangiogenic activity of taxifolin could be due to the inhibition of the autophosphorylation of several tyrosine residues in VEGFR-2 [61].

Carotenoids are C40 tetraterpenoids, a widely distributed group of lipid-soluble pigments found in vegetables and fruits. Olive oil contains several types of carotenoids, with lutein and β-carotene being the main compounds present. The antiangiogenic potential of β-carotene has been demonstrated by means of the rat aorta ex vivo and murine in vivo assays. This pigment has been described to inhibit—in vitro—endothelial proliferation, migration, and tube formation. β-Carotene downregulates the expression of MMP-2, MMP-9, prolyl-hydroxylase, and lysyl-oxidase, and upregulates the expression of the tissue inhibitors of metalloproteinases TIMP-1 and TIMP-2 [84]. Dietary lutein inhibits mammary tumor growth by induction of apoptosis in tumor cells, and inhibition of angiogenesis [85]. Since lutein/zeaxanthin is the eye macular pigment, an adequate intake of lutein-rich foods could also be recommended to the general population in order to reduce the progression of age-related macular eye disease and cataracts [86].

Vitamin E now refers to eight different isoforms that belong to two categories, tocopherols (four saturated analogues, α, β, γ, and δ), and tocotrienols (four unsaturated analogues), with all these compounds beiing found in virgin olive oil [87]. Tocotrienol, but not tocopherol, inhibits both the proliferation and tube formation of BAECs. The most active compound is δ-tocotrienol, which inhibits proliferation, migration, and tube formation of human endothelial cells at low micromolar doses, whereas similar doses of α-tocopherol did not show any effect [88,89]. Mechanistically, downregulation of VEGF expression, suppression of VEGFR-2 signaling, and activation of apoptosis in endothelial cells could be behind the antiangiogenic activity of this compound, confirmed in vivo by the CAM and the Matrigel plug assays [87,89,90].

Although less characterized, a putative antiangiogenic potential for the acyclic diterpenoid phytol, and campesterol and β-sitosterol—two components of sterol fraction of olive oil—has been suggested [91,92,93].

## 5. Antiangiogenic Polyphenols from Fruits and Red Wine

**Figure 3:**
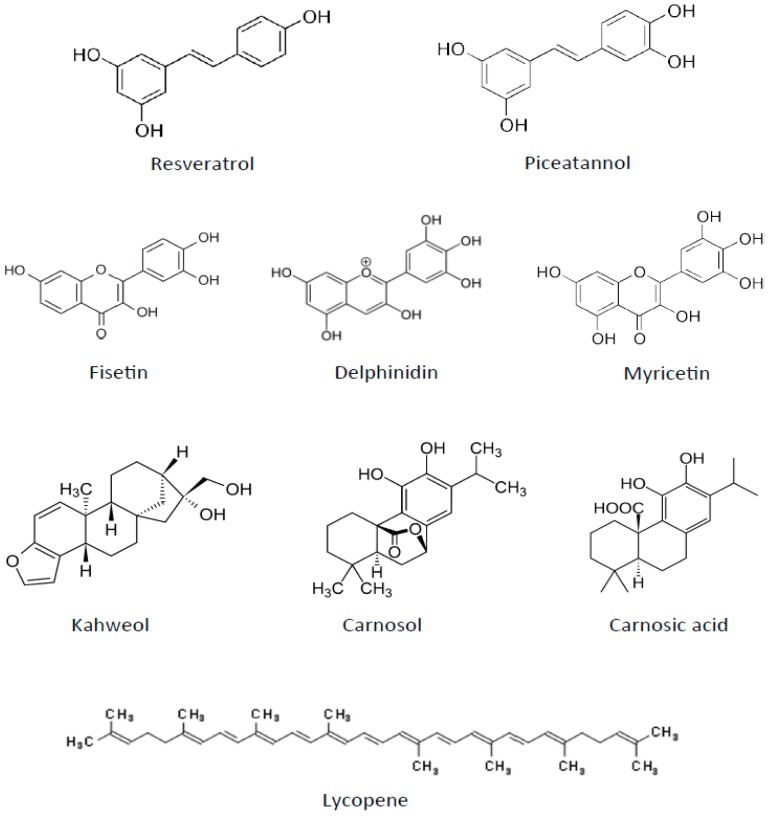
Chemical structures of other antiangiogenic compounds present in the Mediterranean diet (MD) components.

The MD is characterized by a high consumption of fruits and vegetables. It may also include having a glass of wine with lunch every day—mainly red wine, which has been shown to exert a positive influence upon health. Several non-alcoholic components of wine, mainly polyphenols, which are also present in grapes and other fruits, may be responsible for a considerable part of the reported cardioprotective effects of a moderate wine intake [94]. Red wine polyphenolic compounds also exhibit antitumoral activities, which could be, at least in part, mediated by their antiangiogenic activity [95]. Some of these compounds are presented here (Figure 3).

### 5.1. Resveratrol

Resveratrol (3,4′,5-trans-trihydroxystilbene) is a non-flavonoid polyphenol contained in the skin of red grapes, but also found in peanuts and berries. It is probably the most studied red wine polyphenol, and it presents a clear antiangiogenic activity mediated by the inhibition of VEGF expression in tumor cells and by suppressing the endothelial cell response to this angiogenic factor [96,97]. Unlike this antiangiogenic role of resveratrol in cancer, this compound has been described as proangiogenic in some diseased contexts with defective vascularization [98,99,100]. This dual effect of resveratrol could be due to a dose-response activity, since high concentrations of resveratrol induce angiogenesis, whereas low doses are antiangiogenic [101]. The exact mechanisms of the antiangiogenic activity of this compound are not yet completely unraveled, although the modulation of the VEGF/VEGFR-2 pathway through several axes (HIF-1α and GSK3b/β-catenin/TCF) could be in the basis of this effect [102,103,104]. In vitro studies show that trans-resveratrol is the most active stereoisomer [105]. Because of the pharmacological interest of resveratrol, derivatives with improved antiangiogenic activity have been synthetized [106].

### 5.2. Piceatannol

Piceatannol (3,3′,4,5′-tetrahydroxystilbene), a natural occurring stilbene found in grapes and red wine, is a metabolite derived from resveratrol through the activity of the cytochrome P4501B1 enzyme [107]. This compound confers cardiovascular disease protection, preventing atherosclerosis [108], as well as exhibiting anti-aging, anti-inflammatory, anti-diabetic, and anti-tumoral activity [109]. Focusing on its antiangiogenic effects, piceatannol was found to inhibit the in vitro tubular-like structure formation by endothelial cells, most likely by the inhibition of the spleen tyrosine kinase [110]. Piceatannol was also found to decrease the levels of some pro-angiogenic and pro-lymphangiogenic factors, such as VEGF-A, VEGFR-2, VEGF-C, and LYVE-1, through modulation of NF-κB and STAT3 transcription factors in breast cancer in vivo, and diminished macrophage infiltration in tumor tissue via attenuating the MCP-1 and M-CSF chemoattractants [111].

### 5.3. Fisetin

Fisetin (3,3′,4′,7-tetrahydroxyflavone) is a bioactive flavonol found in wine, as well as in several fruits (strawberries, apples, mangoes, persimmons, kiwi, and grapes), vegetables (tomatoes, onions, and cucumbers) and nuts. This compound exhibits a multiple bioactive role in chronic diseases, showing anti-inflammatory, antidiabetic, antioxidant, antitumorigenic, antiinvasive, antiangiogenic, neuroprotective, and cardioprotective effects [112]. The inhibition of in vivo and in vitro angiogenesis by this compound suggests its angiopreventive potential [113]. Fisetin has been reported to decrease ADAM9, a disintegrin and metalloproteinase implied in tumorigenesis and angiogenesis, with this reduction being related to the inhibition of migration and invasion in glioma cells [114]. Indeed, fisetin diminishes VEGF expression in lung adenocarcinoma cells via a HIF-1α independent mechanism [115]. Inhibition of migration and invasion has also been seen in melanoma cells through reduction of MAPK and NFκB signaling pathways, and could be related to the observed disruption of angiogenesis in xenograft melanoma tumors [116,117]. However, the effects of fisetin are not exclusive to tumor cells, since this flavonoid causes microtubule stabilization in endothelial cells [118], that could be, at least partially, the cause of the observed inhibition of migration and tube formation of endothelial cells in vitro, and of the reduction on microvessel density in lung adenocarcinoma tumors [119].

### 5.4. Delphinidin

The antiangiogenic activity of delphinidin (3,3′,4′,5,5′,7-hexahydroxyflavylium), an anthocyanin present in red wine and in many pigmented fruits and vegetables, has been clearly demonstrated by a number of in vitro assays showing inhibitory effects on proliferation, migration, and tube formation by endothelial cells. This activity was confirmed in vivo by the CAM and the Matrigel plug models [120,121]. This activity has been linked to a blockade of VEGF and PDGF-signaling pathways [121,122], although recent results have indicated that it could be also mediated by inhibition of HIF-1α and VEGF expression in tumor cells [123].

### 5.5. Myricetin

Myricetin (3,3′,4′,5,5′,7-hexahydroxyflavone) is a flavonoid found in red wine and grapes, onions, and berries, with an interesting potential in chemoprevention of skin cancer [124]. With a similar chemical structure to that of delphinidin, its role as an angiogenesis inhibitor has been studied in different cancer types, being reported to inhibit the PI3-K/Akt pathway, both in tumor and endothelial cells [125]. Moreover, in a mouse model of skin tumorigenesis, myricetin inhibited UVB-induced angiogenesis, decreasing VEGF, HIF-1, MMP-2, MMP-9, and MMP-13 expression [126,127].

## 6. Other Components of the MD with Angiopreventive Potential

### 6.1. Fish

One of the characteristics of the MD is the high intake of oily cold-water fish, including sardines, tuna, mackerel, and anchovies. These all are known to contain high amounts of omega-3 fatty acids—long-chain polyunsaturated fatty acids with the first double bond three carbons from the methyl end of the chain. Humans cannot desaturate this double bond, and hence omega-3 fatty acids are essential and must be included in the diet, with their consumption being advisable instead of that of omega-6 fatty acids in order to prevent the onset of several types of cancer [128,129,130].

The beneficial effects of omega-3 fatty acids could be related to their inhibitory effects on angiogenesis, either by a direct antiangiogenic activity, or by a reduction of the levels of angiogenic factors and their receptors. In this regard, eicosapentanoic acid and derivatives have been shown to inhibit some angiogenic characteristics, such as tube formation, migration, metalloproteinase expression, and VEGFR-2 activation in vitro [131,132]. Docosahexanoic acid derivatives were also able to inhibit angiogenesis in vitro and in vivo [133]. Enzymes from the arachidonic acid metabolic pathway seem to be essential for the antiangiogenic activity of polyunsaturaled fatty acids [134,135]. In addition, dietary omega-3 diminished VEGF levels in several in vivo models of cancer [136], which is in agreement with the decreased levels of circulating VEGF observed in healthy volunteers after an omega-3 rich diet [137]. Furthermore, beneficial effects of omega-3 fatty acids in rats with 2-4-6-trinitrobenzen sulfonic acid (TNBS) induced colitis has been related to the downregulation of VEGFR-2 [138].

### 6.2. Tomatoes

As a consequence of Christopher Columbus’s voyages, novel foods from the New World were incorporated into the MD, including tomatoes (*Solanum lycopersicum*). Nowadays, tomatoes are essential in Mediterranean cuisine, being highly consumed either raw, in salads, or in cold soups such as the refreshing and tasty Andalusian gazpacho, or cooked as an essential ingredient of sauces and other meals. Increasing evidence supports a correlation between tomato consumption with a reduced risk of cardiovascular diseases and cancer [139,140]. Tomato is an important source of vitamin C, potassium, folic acid, and carotenoids, such as lycopene. The postulated cancer chemopreventive potential of lycopene [141,142,143,144] (Figure 3) could be reinforced by the use of this compound in angioprevention. Lycopene inhibits angionesis in vitro and in vivo at a concentration that should be achievable by dietary means [145]. This activity has been related to the MMP-2/uPA system inhibition through VEGFR-2-mediated PI3K-Akt and ERK/p38 signaling pathways [146]. Aside from lycopene, cystine-knot miniproteins present in tomato fruit (TCMPs) are now being postulated as useful inhibitors of angiogenesis. These proteins, present in mature tomato fruits, display resistance to gastrointestinal proteolytic attack and resistance to industrial processing [147]. Their antiangiogenic activities, demonstrated in vitro by the inhibition of endothelial tube formation and migration, as well as in vivo by using a zebrafish model, suggest that these proteins, along with lycopene and other antioxidants, may confer beneficial effects to tomato dietary intake [148,149].

### 6.3. Dairy Products

At a low level, the consumption of dairy products is included in the MD. These dairy products are, according to the ancient MD, mainly yoghurt (usually strained, a procedure that decreases lactose and increases protein content) and soft white fermented cheese (feta cheese in Greece or myzithra in Crete), with full-cream goat and sheep milk consumption being reserved for children [150]. Some components of milk have been studied in the context of angiogenesis modulation and chemoprevention of cancer, especially proteins and peptides [151,152], suggesting that milk components could play a role in angiogenesis inhibition and hence in angioprevention. In particular, the bovine milk proteins lactoferrin and lactoferricin (a pepsin-generated peptide derived from lactoferrin) have been described as angiogenesis inhibitors by using in vivo tumor models and in vitro approaches [153,154,155]. Mechanistically, both proteins seem to exert a similar activity by inhibiting the VEFG-A165-mediated angiogenesis in vivo [155,156]. Although these milk components support the potential role of dairy products in angioprevention, this property is not still well defined, as other proteins present in milk have shown proangiogenic activity. In particular, angiogenin-2, present in bovine milk and serum, has shown a proangiogenic effect in vivo using the CAM model [157]. Lactadherin (also known as MFG-E8 (milk fat globule epidermal growth factor-8)), another bioactive milk protein, has been described as a promoter of VEGF-dependent angiogenesis, exhibiting a protumoral role in different in vivo models [158,159,160].

### 6.4. Beverages

Attending to beverages included in the MD, apart from the daily consumption of water and the low intake of red wine within principal meals, the moderate consumption of coffee and herbal tea represents an important part of the MD [150]. Coffee contains a high variety of bioactive compounds, such as caffeine, chlorogenic acid, diterpenes, alkaloids, and polyphenols. Focusing in the potential role of coffee as an angiopreventive beverage, some of these compounds have shown its ability to inhibit angiogenesis [161,162]. This is the case of kahweol (Figure 3), an antioxidant diterpene present in coffee beans and unfiltered coffee beverages. Kahweol has been shown to inhibit key steps of angiogenesis in vitro, showing a potent antiangiogenic activity ex vivo (mouse aortic ring assay) and in vivo (CAM and zebrafish intersegmental vessel models) [161]. It also targeted inflammatory molecules such as COX-2 and MCP-1 in endothelial cells. Interestingly, kahweol palmitate, the diterpene ester derived from kahweol, maintained the antiangiogenic properties of kahweol in vitro, pointing to a mechanism of action that involves downregulation of the Akt pathway through negative modulation of VEGFR-2 [162]. Coffee is a highly consumed beverage worldwide, but the content of kahweol and other bioactive compounds is variable depending on the type of coffee and its processing (filtering of coffee is a critical step in affecting the content of kahweol). For this reason, it is important to remark that traditionally, the coffee included in the MD is the boiled (unfiltered) Greek coffee, which is rich in kahweol, as well in polyphenols and other antioxidants, and containing moderate amount of caffeine [163,164]. Indeed, in the interesting Ikaria study, the consumption of this type of coffee was associated with improved endothelial function [165].

### 6.5. Nuts

Other sources of bioactive compounds in the MD that are relevant to angioprevention are nuts. The MD includes mainly almonds, hazelnuts, walnuts, pistachios, and pine nuts, which are consumed moderately in the form of snacks, as part of cooked meals, or as nut-based desserts. The content in nutrients and phytochemicals in nuts is variable, but in general these foods are rich in unsaturated fats, vitamin B (folate), vitamin E (tocopherols), and polyphenols [166]. Among the key MD nuts, walnuts are especially relevant for angioprevention, as they represent a very important source of both linoleic and α-linolenic acid (omega-3 fatty acids) and contain significant amounts of the aforementioned inhibitor of angiogenesis—tocopherol. The effect of linoleic acid on angiogenesis modulation is contradictory, since both pro- and antiangiogenic roles have been described for this fatty acid in several models [167,168,169,170]. An interesting study showed that in vivo and in vitro treatments with linoleic acid produced an increase in the DNA methylation level of the VEGF-B promoter, which translated into lower levels of expression of the VEGF-B gene [171].

### 6.6. Seasonings

Traditional seasoning in the MD includes olive oil and herbs/spices. Although the quantity of herbs as a part of a diet is low, in the context of the MD, these traditional seasonings exhibit an interesting potential as a natural source of compounds able to inhibit angiogenesis. One of the most commonly used seasoning herbs in the MD is dry oregano (*Origanum vulgare*), which contains polyphenols in a high proportion [172]. Of note, the antiangiogenic effect of an ethanol extract of oregano (containing mainly phenolic acids and flavonol derivatives) was assessed in vivo and in vitro in the context of breast cancer, showing a decrease in the expression of VEGFR-2 in tumour cells [173]. Another example of a popular herb used in MD seasoning is rosemary (*Rosmarinus officinalis*), containing carnosol and carnosic acid, two diterpenes that have been described as inhibiting angiogenesis in vitro and in vivo by a mechanism that implies apoptosis induction in endothelial cells [174,175].

## 7. Conclusions

The scientific evidence pointing to the deep influence of diet on health involves an important change in biomedical research and clinical practice. Nutrients and bioactive compounds contained in foods have become essential players in numerous diseases at multiple levels, from initiation to progress, opening the possibility of studying the preventive and therapeutic role of certain elements of diet and even of specific dietary patterns. However, perhaps the main implication arising from this evidence is that the general population, to a certain extent, can control by themselves their own health easily by adapting their diet to a healthier model and consuming foods with bioactive compounds that could prevent the onset of certain diseases. A better understanding of how the genetic makeup of an individual modulates his/her differential response to nutrients, identifying and characterizing the gene–diet interactions, could help to optimize the individual dietary choices aimed at increasing quality of life, health, and longevity [176]. The use of integrative nutritional biomarkers could facilitate the achievement of “precision nutrition”, understood as personalized dietary recommendations that could produce optimal health effects in particular individuals [177].

In this context, the MD emerges as an interesting dietary pattern because of the reported beneficial effects of this diet on numerous pathologies, mainly cardiovascular diseases, but also diabetes, neurodegenerative diseases, and cancer [43]. The MD represents an important source of biologically active compounds and the preventive and therapeutic role of several of them in specific pathologic contexts constitutes a large subject to study. Aside from the interesting individual effects of the bioactive components of the MD, it is important to remark that these compounds usually are low-dose components of food, so it is feasible to argue that the preventive benefits of the MD on certain diseases could reside in the combination of foods containing different bioactive compounds that row in the same direction. This idea is reinforced by the fact that these diet-derived compounds exhibit relative non-toxic and safety profiles; hence, the long-term consumption of several of them in foods at the same time would rarely represent a risk.

**Figure 4:**
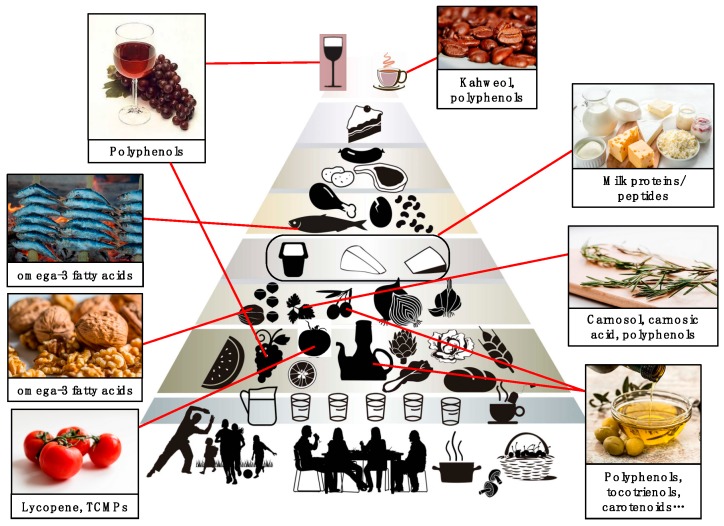
The Mediterranean diet (MD) as a source of antiangiogenic compounds with potential use in cancer angioprevention. Schematic representation of the MD pyramid (modified from Fundación Dieta Mediterránea [27]), showing the main components that could have a role in angioprevention due to their content in antiangiogenic bioactive compounds.

Angioprevention, and specifically diet-mediated angioprevention, is an emerging concept with important consequences for the prevention of cancer and other angiogenesis-dependent pathologies [22]. The link between angioprevention and MD has been minimally explored, even though there is clear implication of several MD components in angiogenesis inhibition, as reviewed here, which is summarized in Figure 4. The implementation of the MD concept at the three levels of cancer prevention would require in turn three levels of management (Figure 1). At the first level, adopting a high adherence to a MD would imply a certain level of angioprevention in the general population; at the second level, supplementation of the MD with specific purified components or even nutraceuticals would lead to a reinforced preventive effect on the risk population; and finally, in the third level, either isolated MD bioactive molecules or their derivatives could be administered as drugs to increase therapy efficacy, diminish side effects, or enlarge the disease-free time of cancer patients. Prevention is probably the most cost-effective long-term anticancer strategy. With increasing frequency, cancer becomes a chronic disorder for many patients, who must be treated for a long time. In this context, angiopreventive strategies based on the MD components could help to control the disease, with reasonable cost and minimizing long-term toxicities.

In parallel to the growing evidences of the benefits of the MD in health, in recent years, a worrisome tendency towards a western diet in Mediterranean countries has been assessed, with clear possible implications in the general health status [29,30]. Hence, the importance in increasing the current adherence of the population to the MD is a crucial point in disease prevention. Present and future research about the molecular basis of the angiopreventive role of the MD could help to promote the return to a healthier way of life.

## 8. Future Perspectives

The Mediterranean diet arises as a rich and varied source of natural antitumoral and antiangiogenic compounds, pointing to a relevant chemopreventive potential that should be confirmed by clinical studies. For the general population, increasingly interested in the self-control of health, the knowledge of angiopreventive strategies based in consumption of selected foods should help to promote healthier dietary habits, preventing the initiation and/or progression of cancer. Personalized angiopreventive programs would be of useful application to the population with a high risk of developing cancer, for which improvement in the screening programs and the diagnostic tests are needed. Finally, cancer is becoming, with an increasing frequency, a chronic disorder for many patients, who must be treated for a long period of time. In this context, angiopreventive strategies based on MD components could help to control the disease with reasonable cost and minimizing long-term toxicities.

## Figures and Tables

**Table 1:** Antiangiogenic drugs approved by Food and Drug Administration (FDA)/ European Medicines Agency (EMA) for the treatment of solid tumors. Referenced from [13,14,15].

Drug	Type	Molecular Target	Malignancies
Axitinib (Inlyta)	TKI	VEGFR-1-3, PDGFRβ	Advanced RCC
Bevacizumab (Avastin)	Humanized monoclonal antibody	VEGF	MCRC, NSCLC, OC, MBC, glioblastoma, metastatic RCC, endometrial cancer, Mesothelioma, and cervical cancers
Cabozantinib (Cometriq)	TKI	VEGFR-2, Tie2	Refractory advanced RCC, metastatic medullary TC, and pancreatic neuroendocrine tumors
Cediranib	TKI	VEGFR-1-3	OC
Everolimus (Afinitor)	TKI	mTOR	RCC, gastrointestinal cancer, lung carcinoma, advanced breast cancer, pancreatic neuroendocrine tumors (PNETs), and subependymal giant cell astrocytoma
Lenalidomide (Revlimid)	Amino acid	VEGF, bFGF	Myeloma (myelodysplastic syndrome (MDS)) and mantle cell lymphoma
Levantinib (Lenvima)	TKI	VEGFR-1-3, PDGFRα, FGFR-1-4	TC, HCC, and RCC
Pazopanib (Votrient)	TKI	VEGFR-1-3, PDGFRβ, FGFR-1-2	Metastatic STC and advanced RCC
Ramucirumab (Cyramza)	Human monoclonal antibody	VEGFR-2	MCRC, NSCLC, and gastric adenocarcinoma
Regorafenib (Stivarga)	TKI	VEGFR-1-3, PDGFRβ, FGFR-1-2	Chemo-refractory MCRC, unresectable HCC, and GIST
Sorafenib (Nexavar)	TKI	VEGFR-2, PDGFRβ	Advanced RCC, metastatic differentiated TC, and unresectable HCC
Sunitinib (Sutent)	TKI	VEGFR-1-2, PDGFRσ/β	Metastatic RCC, gastrointestinal stromal tumors, and pancreatic neuroendocrine tumors
Thalidomide (Synovir, Thalomid)	Amino acid	VEGF, bFGF	Multiple myeloma
Temsirolimus (Torisel)	TKI	mTOR	RCC
Vandetanib (Caprelsa)	TKI	VEGFR-2	Unresectable or metastatic TC
Ziv-Aflibercept (VEGF-Trap) (Zaltrap)	Fusion protein (VEGFR chimera)	VEGF-A/B, PlGF	MCRC

VEGF (vascular endothelial growth factor), VEGFR (vascular endothelial growth factor receptor), bFGF (basic fibroblast growth factor), FGFR (fibroblast growth factor receptor), PDGFR (platelet-derived growth factor receptor), mTOR (mammalian target of rapamycin), PlGF (placental growth factor), TKI (tyrosine kinase inhibitor), MCRC (metastatic colorectal carcinoma), NSCLC (non-small cell lung cancer), OC (ovarian cancer), MBC (metastatic breast cancer), RCC (renal cell carcinoma), HCC (hepatocellular carcinoma), GIST (gastrointestinal stromal tumor), TC (thyroid carcinoma), STC (soft tissue carcinoma).
